# SAAQ: A Characterization Method for Distributed Servers in Ubicomp Environments

**DOI:** 10.3390/s22176688

**Published:** 2022-09-04

**Authors:** David Ferere, Irvin Dongo, Yudith Cardinale

**Affiliations:** 1 Departamento de Telecomunicaciones, Universidad Simón Bolívar, Caracas 1080, Venezuela; 2 Electrical and Electronics Engineering Department, Universidad Católica San Pablo, Arequipa 04001, Peru; 3 ESTIA Institute of Technology, University Bordeaux, 64210 Bidart, France; 4 Escuela Superior de Ingeniería, Ciencia y Tecnología, Universidad Internacional de Valencia, 46002 Valencia, Spain

**Keywords:** middleware, load balancing, Ubicomp, IoT

## Abstract

The increasing evolution of computing technologies has fostered the new intelligent concept of Ubiquitous computing (Ubicomp). Ubicomp environments encompass the introduction of new paradigms, such as Internet of Things (IoT), Mobile computing, and Wearable computing, into communication networks, which demands more efficient strategies to deliver tasks and services, considering heterogeneity, scalability, reliability, and efficient energy consumption of the connected devices. Middlewares have a crucial role to deal with all these aspects, by implementing efficient load balancing methods based on the hardware characterization and the computational cost of the queries and tasks. However, most existing solutions do not take into account both considerations in conjunction. In this context, we propose a methodology to characterize distributed servers, services, and network delays in Ubicomp environments, based on the Server Ability to Answer a Query (SAAQ). To evaluate our SAAQ-based methodology, we implemented a simple middleware in a museum context, in which different IoT devices (e.g., social robots, mobile devices) and distributed servers with different capabilities can participate, and performed a set of experiments in scenarios with diverse hardware and software characteristics. Results show that the middleware is able to distribute queries to servers with adequate capacity, freeing from service requests to devices with hardware restrictions; thus, our SAAQ-based middleware has a good performance regarding throughput (22.52 ms for web queries), end-to-end delay communications (up to 193.30 ms between San Francisco and Amsterdam), and good management of computing resources (up to 80% of CPU consumption).

## 1. Introduction

The introduction of advanced paradigms, such as Internet of Things (IoT), Mobile and Wearable computing, into communication networks has set the Ubiquitous computing (Ubicomp) as the new era in the history of computing technologies [1]. Ubicomp environments allow objects and people to be connected anytime and anywhere, to anything and anyone, by any-network on any-service [2]. Therefore, many applications in many areas have been developed in order to allow different kinds of devices and technologies to communicate with each other and integrate them into one ecosystem [3]. Consequently, achieving interoperability of heterogeneous networks becomes a complex challenge [4], that demands the development of integration systems to provide efficient and fast communications among all Ubicomp network instances.

In Ubicomp systems, any device connected to the network could act as a server in some specific scenarios (e.g., sensor interchanging data in a Wireless Sensor Network, a robot sending data to another one); thus, any query could be responded by any client/server in the Ubicomp ecosystem. For that, the design of efficient scheduling and dispatcher mechanisms able to distribute the service requests is crucial. These mechanisms have to take into account different factors related to the computational capabilities of all instances of the network (hardware configuration of devices) and the computational resources demanded to satisfy the request (complexity of the query). For instance, processing image queries, which require high computational power [5,6], cannot be assigned to a robot because it has low computational capabilities, and its tasks should be optimized for saving energy.

Middlewares, as intermediate pieces of software, have appeared as solutions to manage such heterogeneity, scalability, and interoperability issues in Ubicomp environments [7]. A middleware abstracts the vast of different technologies and communication protocols by integrating them into a universal communication layer [3]. In the context of IoT systems, among the communication middlewares that have been developed, there are general open-source solutions, such as OpenIoT [3,8], OpenRemote [9], Kaa [10], Xively [11], and ThingBroker [12], and others have been proposed and applied in specific fields, such as e-health [13], smart cities [8,11], early fire detection [10,14], water supply [9,10], intelligent car parking [15], agriculture [10], home appliances [9,10], and museums [4]. However, existing general and customized middlewares do not take into account either the server capabilities and energy saving needs or the computational cost of the queries. Non-efficient distribution of requests can lead to the wasting of resources by assigning and making busy high-performance servers deal with simple tasks, such as web information requests, or to the overcharging of devices with low computational capacities and battery restrictions by dispatching them for high power computing tasks.

To overcome these limitations, we propose a methodology to characterize distributed servers, services, and network delay, based on the **S**erver **A**bility to **A**nswer a **Q**uery (SAAQ). This SAAQ-based methodology is defined in two phases:

- The **configuration phase** comprised of characterization methods to represent: (i) servers, considering CPU, RAM, and GPU capabilities; (ii) queries, considering CPU, RAM, and GPU requirements; and (iii) the network delay.
- The **assignment phase** which manages: (i) an SAAQ score, that represents the result of calculating capabilities of servers with respect to queries and network delay; and (ii) a distribution query process, considering the SAAQ score values.

The distribution process assigns queries to the servers with the minimum resources that satisfy the queries in order to save resources for more complex queries, and thus, an adequate energy management. By following this methodology, a query can be assigned to a specific server according to the computational cost of the query and the computational capabilities of the server, as well as the network delay. To validate our method and show its suitability, we integrate this methodology into a simple middleware in a museum context in which different IoT devices (e.g., social robots, mobile devices) and distributed servers of different capabilities can participate. We defined six queries in this context with different computational costs and let the middleware perform workload balancing, considering different scenarios related to server capabilities and client locations. Results show that considering our SAAQ-based methodology, local servers (temporal/permanent IoT devices) obtained up to 80% maximum use of CPU, since they are the most suitable servers due to the impact of network delay on response times. Moreover, experiments demonstrate the remote servers close to clients improve response times. Our SAAQ-based middleware has a good performance regarding throughput, end-to-end delay communications, and good management of computing resources.

The remainder of this paper is organized as follows. Section 2 presents a motivating scenario on the need to develop a characterization method for distributed servers in Ubicomp environments. In Section 3, we survey relevant and recent related work. In Section 4, our characterization method is described. Section 5 reports the experimental evaluations and the obtained results. Finally, in Section 6, we conclude the paper with some considerations and future work.

## 2. Motivating Scenario

In order to illustrate the motivation behind the design and implementation of a methodology for load balancing among distributed servers and IoT devices in a Ubicomp context, let us consider a museum scenario, where several types of devices (e.g., robots, smartphones, computers) generate different types of requests (e.g., image processing, web-page information, robot location). In this context, internal and external devices are considered, such as a robot in the museum (internal device), which consumes location and image processing services, and a tourist personal device (external device), which accesses services of a tourism information web page. When an internal device requires information, it acts as a client, but it also can act as a local server in some cases. For example, a robot (local device) which has location information about a room, can provide this information to other robots (other local devices) for a fast route planning. Note that a robot uses limited range sensors (e.g., cameras) in order to map the rooms, and this task requires time to be completed.

**Figure 1:**
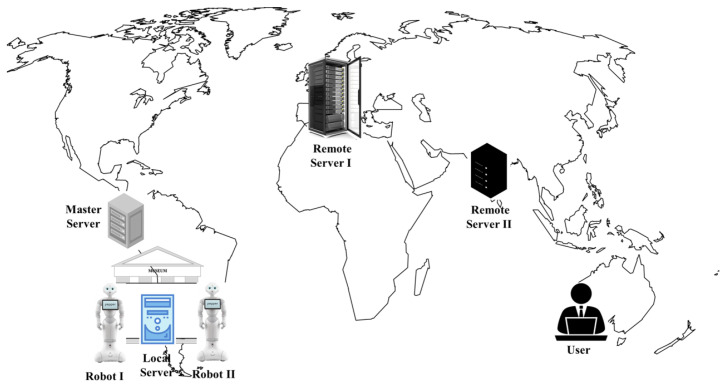
A museum scenario with IoT devices.

Figure 1 shows our motivating scenario, which contains: (i) a medium-performance server (external device) acting as a Master Server (a common centralized middleware) and located close to the museum; (ii) a local low-performance server (Local Server) in the museum; (iii) a remote high-performance server located in another country (Remote Server I); (iv) a remote medium-performance server also located in a different country (Remote Server II); (v) a Robot I and a Robot II in the museum, acting as local devices and clients; and (vi) a user searching for information about the museum (external device), in another country next to a remote server.

Considering that Robot I in the museum (acting as a client) requires image processing for its onsite location, which demands a lot of computational resource [5,6,16,17,18], it is appropriate that a powerful server attend to this request for better response times, taking into account its location due to the delay of the network. Therefore, the remote high-performance server (Remote Server I) responds faster than the local low-performance server even if there is a network delay. Later, Robot II requires the same image processing. Then, since Robot I already made the same query and has the response, the middleware in the Master Server assigns that request to Robot I, acting as server in this scenario.

In the case of the external client (User’s device in Figure 1) who makes requests about the museum located at the same country as one of the remote servers, it is adequate that the remote server (Remote Server II) responds to the request to reduce the network delay (if the server is able to perform it).

By this motivating scenario, the following requirements are necessary to ensure a proper load balancing in a Ubicomp environment:

- A **semi-centralized architecture**, where a master medium-high server is able to distribute the requests. The heterogeneity of servers and local-client devices that can become servers for particular cases require a central powerful coordinator to reduce the complexity of the architecture with respect to a fully distributed one, where all entities determine their computational capabilities, wasting resources, especially for low-performance servers.
- **Characterization methods** for servers and requests to determine which servers are the most adequate to attend to a specific type of request based on:
  – The computational capabilities, where the demand of CPU, RAM, and GPU are variable according to the type of requests.
  – The network delay, which has an impact on the response time that in most cases is proportional to distance between the entities [19,20].

Existing open-source [3,21] and customized [4,15,22] middlewares are not suitable for this scenario, since they focus on workloading distribution among the servers and consider the availability as the main goal, i.e., an available low-performance server could be assigned to perform a high-performance-demanding incoming request, increasing the response time, while a high-performance server can be busy with simple tasks at the time a high-performance request comes, being unable to resolve it, increasing once again the response time. In this sense, having a wide and heterogeneous server-farm and traditional workload balancers, low response times are obtained due to a distribution of the workload with respect to a single powerful server, but a correct computational resource management cannot be ensured since a request can be resolved for a random server.

## 3. Related Work

In Ubicomp environments, it is common to find computing architectures where nodes with low capabilities or energy consumption restrictions can behave as clients and servers; thus they receive data and queries from other devices in real time. In this sense, the distribution of tasks is a key aspect to reach efficient global performance as current studies have highlighted [23].

In this sense, many works highlight the main issues that impact the energy consumption savings and efficient workload distribution in Ubicomp environments, such as the large number of heterogeneous devices, the high variability of services’ latency, and the huge amounts of data generated from the connected devices devices, which in turn implies high cost of communication bandwidth and high redundancy of data [15,19,24,25,26,27,28]. In this regard, all these works agree the load balancing solutions for Ubicomp middlewares have to be set on the following considerations:

- Location awareness to allow Ubicomp servers closer to end users to respond to their queries and thus reduce the communication costs.
- Energy awareness to distribute the queries and tasks to devices without energy consumption restrictions, as much as possible.
- Consider the query cost (service characterization) and the capabilities of servers and the network state (hardware characterization) to ensure an efficient management of computational resources, therefore providing the operation of large-scale Ubicomp networks.

Based on these considerations, many studies have proposed middlewares or software components to partially overcome the current challenges. Many works exploit the location awareness; thus they are focused on data management [13,29,30], neglecting the energy consumption awareness and characterization of services and servers.

Some other works tackle the problem of energy awareness for load balancing in Ubicomp environments [31,32,33,34,35], but they do not perform a better distribution considering the capabilities of servers.

Characterization of services is also a strategy used to support decisions about scheduling and distribution of requests in a distributed system. In the context of museums, as our motivating scenario, some works have proposed solutions in this regard. The semantic information service layer described in [36] characterizes services (i.e., visiting, exhibition, and enrichment services) and devices (e.g., multimedia, personal, smart IoT, and network communication) to support the design of intelligent application; however, this information is not used in run-time for task distributions. An IoT architecture system is developed in [4] aimed at transforming Museums and Historical Centres in Smart Places by introducing IoT technologies. They propose a system organized in three layers, a sensors layer, network layer, and application layer, to gather information from the environment and characterize it to decide which services are activated. The study presented in [37,38] categorizes services related to a robot implementation in Smart Museums and Smart Places. Authors propose the use of robots for museum settings and for learning heritage languages and cultures at the Chinese heritage center; for that purpose, they use two social robots, one to guide visitors and the other one for explaining and presenting the artwork (English and Chinese languages). The robots have an architecture framework organized in two layers, a physical layer and a middleware layer, where there are a localization module, a word processing module, a navigation module, and a response content module. Authors of the work presented in [38] talk about the integration between Smart Environments (AmI—Ambiance Intelligence) and Mobile Robots Team (MRT) to increase performance of task execution and human–robot interaction in smart environments such as museums, medical centers, or warehouses. They consider three elements for this integration system: humans, robots, and sensors. The robots belong to a semantic layer. Additionally, there is a scheduling layer that defines the assignment tasks scheme and finally an execution layer that executes all the tasks demanding from robots and sensors.

All these works demonstrate the need and usefulness of better strategies to deal with distributed services in Ubicomp environments, where devices with different levels of capacities are involved in the demand and delivery of services. However, few of them propose holistic solutions that integrate the three previously considered aspects: location awareness, energy awareness, and categorization of services and servers. Thus, a method able to ensure the correct use of resources in Ubicomp environments, is needed.

## 4. SAAQ-Based Methodology: Our Proposal

**Figure 2:**
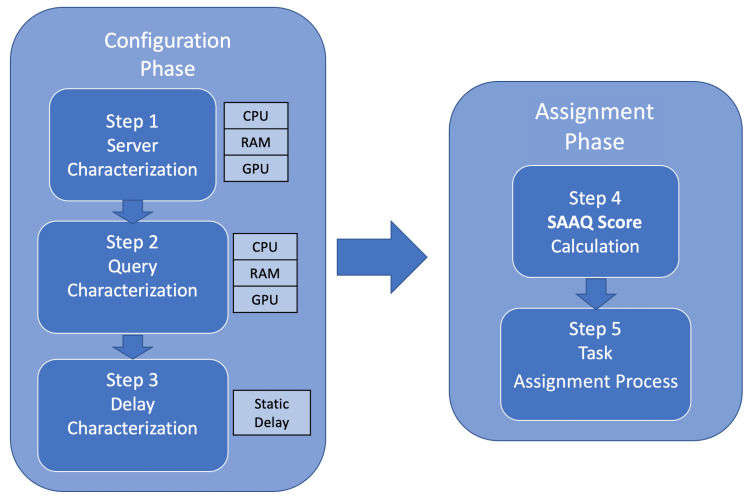
SAAQ methodology pipeline.

According to the issues outlined in Section 3, such as location awareness, energy awareness, and categorization of services and servers, we design a methodology to assign queries to servers available and capable of answering queries in the shortest time. Our proposed SAAQ-based methodology comprises the configuration phase, and the assignation phase, as shown in Figure 2. In the configuration phase servers and queries are characterized by considering three aspects: (i) the computational capabilities of the server in terms of CPU, RAM, and GPU capacities [5], which are the most representative resources of a computer (Step 1 in Figure 2); (ii) the computational cost of the query, defined in terms of CPU, RAM, and GPU needed for a query to be executed in a server (Step 2 in Figure 2); and (iii) the delay time between clients and servers (Step 3 in Figure 2). In the assignment phase, this methodology defines a metric called SAAQ score ($saqq_{score}$) to relate servers, queries, and network delay and indicate how suitable a server is to resolve a particular query (Step 4 in Figure 2) and a distribution query process that assigns queries to a server according to the SAAQ score values (Step 5 in Figure 2). The following subsections describe both phases and their respective steps.

### 4.1. Configuration Phase

This phase comprises three steps to characterize servers, queries, and network delay and offers methods that can be adapted and extended according to the scenario in which they are applied.

#### 4.1.1. Step 1: Sever Characterization Method (Computational Capabilities)

To determine the computational capabilities of the servers, we use benchmarking methods for CPU and GPU components in order to establish rankings for each resource. A benchmarking is an evaluation method for identifying and understanding the causes of a good performance for any process. The four elements considered by benchmarkings are [39]:

- The objective: in our case the goal is to calculate the overall performance of the servers.
- The practice: represent the actions to measure the server performance.
- The resources to evaluate: in this study are CPU, RAM, and GPU.
- The measurement in terms of consumption and execution time of a task.

By calculating a score for each resource ($s_{resource}$), i.e., CPU, RAM, and GPU, which is obtained based on particular characteristics, a ranking ($r_{resource}$) in the scale of 1 to 10 is established.

To propose the CPU ranking ($r_{cpu}$) based on the CPU server score value ($s_{cpu}$), we use the most popular benchmarking, CineBench [18], which is widely used to evaluate computer performance in rendering processes. According to CineBench, the best CPU in the market is the AMD Threadripper 3990X; thus it is established as the top of our ranking. We calculate our score (see Definition 1) using three elements of the CPU: (i) the CPU speed (GHz); (ii) the numbers of cores; and (iii) the number of threads that is supported by each core.

**Definition** **1.** **CPU Score ($s_{\mathrm{cpu}}$).** *Given a server S, its CPU score, denoted as $s_{cpu}$, is calculated by multiplying its CPU speed (GHz), the number of cores, and the number of threads that is supported by each core, as follows:*

$$s_{cpu}\left(S\right)=S.CPU.speed\times S.CPU.cores\times S.CPU.threads$$

By using the $s_{cpu}$ value, the CPU is ranked in a scale from 1 to 10 ($r_{cpu}$). Table 1 shows the ranges of CPU scores of the proposed ranking and the characterization of servers, according to the CPU, for our motivating scenario in a museum. For instance, the CPU Intel Core i7-8650U (2.7 GHz), which has 4 cores and 2 threads per core, which means in total the CPU can solve 8 threads simultaneously, obtains a CPU score value of 86.4. ($s_{cpu}=2.7\times 4\times 8=86.4$) and therefore classified as $r_{cpu}=2$ in our ranking.

For the RAM, we define the RAM score and ranking based on some recommendations posted in [40,41,42,43]; these works consider most of the queries can be satisfied with less than 16 GB. We define the RAM score ($s_{ram}$) using the amount of RAM (see Definition 2).

**Definition** **2.** **RAM Score ($s_{\mathrm{ram}}$).** *Given a server S, its RAM score, denoted as $s_{ram}$, is calculated by its amount of RAM, as follows:*

$$s_{ram}\left(S\right)=S.RAM.size$$

Table 2 describes our proposed RAM ranking and the characterization of servers, according to the RAM, for the museum of our motivating scenario; for example, if the server has 6 GB of RAM ($s_{ram}=6$), then it is ranked as $r_{ram}=3$.

For GPU, we take into account the UserBenchmark [44] as a reference in order to build the GPU raking ($r_{gpu}$). UserBenchmark has a classification of 652 GPUs in the market. The GPU score ($s_{gpu}$) is calculated by multiplying the speed and the amount of memory (see Definition 3).

**Definition** **3.** **GPU Score ($s_{\mathrm{gpu}}$).** *Given a server S, its GPU score, denoted as $s_{gpu}$, is calculated by multiplying its speed and the amount of memory:*

$$s_{gpu}\left(S\right)=S.GPU.speed\times S.GPU.size$$

In Table 3, we describe the GPU score ranges and the ranking from 1 to 10, as well as the characterization of servers according to the GPU characteristics for our motivating scenario in a museum. For instance, the Nvidia RTX 3090 GPU has 1.70 GHZ speed and 24 GB of memory, then its GPU score is 40.80 ($s_{gpu}=1.70\times 24=40.80$) and according to our scale, it is ranked as $r_{gpu}=10$.

Note that we adopt simple CPU and GPU score calculations based on some characteristics of the resources (e.g., speed, RAM, cores, threads), which are easily obtained by executing commands such as sar, free, and lscpu for Linux; and systeminfo and “WMIC CPU Get DeviceID, NumberOfCores, NumberOfLogicalProcessors” for Windows, and to be calculated at real time. Some servers have low computational capabilities and are battery-energy dependent; therefore running benchmarking tools which nowadays have been adapted to thermal throttling analysis (e.g., 10 min for Cinebench R23 [45,46]) are not suitable for this scenario.

Once the rankings for CPU ($r_{cpu}$), RAM ($r_{ram}$), and GPU ($r_{gpu}$) are defined, our server characterization score based on these values is formalized in Definition 4.

**Definition** **4.** **Server Characterization Score ($c_{\mathrm{score}}$).** *Given a server S, its server characterization score, denoted as $c_{score}$, is defined as a 3-tuple, consisting of its $r_{cpu}$, $r_{ram}$, and $r_{gpu}$ values:*

$$c_{score}\left(S\right)=<S.r_{cpu},S.r_{ram},S.r_{gpu}>$$

Table 4 shows the characterization scores ($c_{score}$) of the servers presented in the museum of our motivating scenario. According to our rankings, Remote Server I has the highest score $c_{score}=<10,10,10>$, while Robot I and Robot II have the lowest value, $c_{score}=<1,2,1>$.

#### 4.1.2. Step 2: Query Characterization Method (Computational Cost)

The types of queries that might be contemplated are strongly related to the domain, context, or scenario in which the Ubicomp system is developed. To illustrate this step, we consider several works related to type of queries and services [15,38,47] and our motivating scenario, defining four categories described as follows:

- Multimedia services, referred to all binary format data like images, videos, and voice recording.
- Localization services, such as services like devices Geo localization, GPS, places description, and environment recognition.
- Web services, meaning all services that allow accessing web applications, webpages, API (Application Programming Interfaces), and cloud platforms.
- Information Management services that manage all personal data, devices metadata and place information stored in DB (Data Bases); therefore, it defines how these data are shared, saved, and transmitted through the middleware.

The query characterization score describes the resources required to perform a task in terms of CPU, RAM, and GPU, similar to the sever characterization score in order to compare both in the SAAQ score calculation. Definition 5 formally presents our query characterization score ($q_{score}$).

**Definition** **5.** **Query Characterization Score ($q_{\mathrm{score}}$).** *Given a query Q, the query characterization score, denoted as $q_{score}$, is defined as a 3-tuple consisting of the minimum power of CPU, RAM, and GPU resources (in a rank of 1 to 10) required to complete the query successfully:*

$$q_{score}\left(Q\right)=<Q.r_{cpu},Q.r_{ram},Q.r_{gpu}>$$

For Multimedia services, we consider image processing queries demanding: (i) GPU Nvidia Quadro 2000 with 1024 MB and 1250 GHz; (ii) CPU Intel Core i7 – 8700; and (iii) 16 GB of RAM memory, as shown in [6,16,48]. Then, according to our server characterization score, we determine that an image processing query demands $q_{score}=<6,5,6>$. In Web services, queries demand real-time interaction, quick response time, and effective communication with cloud servers [49]; therefore, we establish a cost of $q_{score}=<6,4,4>$. In the case of Information Management services, we establish a cost of $q_{score}=<6,5,2>$ for Word Processing queries (less GPU is required), while for Synchronization queries (updating data) the cost is $q_{score}=<1,1,1>$. For Localization services, there are two types of requests: localization by IP address and localization by images. We determine that a localization by image query demands $q_{score}=<6,4,5>$ and by IP address is $q_{score}=<3,2,1>$, according to the studies proposed in [50,51] that characterize images and forest maps analysis to localize firefighters by sensors.

Table 5 summaries the most common services, type of queries, and the query characterization score of six queries, which are presented in applications such as museums and place of interest, similar to our motivating scenario.

#### 4.1.3. Step 3: Delay Characterization (Network Cost)

The delay between servers has an impact on the response time, which in some cases can be bigger than the processing time [52,53]. Based on the work defined in [20], we establish a rank between 0 and 10. Table 6 shows the scores for different delays, our proposed ranking, and the characterization of servers according to the network delay. For example, in our motivating scenario, a delay of 130 ms ($s_{delay}=130$) corresponds to a value $r_{delay}=6$ in our ranking. The delay characterization score is formally defined in Definition 6.

**Definition** **6.** **Delay Characterization Score ($d_{\mathrm{score}}$).** *Given a server S and a query Q, the delay characterization score, denoted as $d_{score}$, is defined as the $r_{delay}$, which is a value in a rank of 0 to 10 according to the network delay between S and S, i.e., $s_{delay}\left(S,Q\right)$:*

$$d_{score}\left(S,Q\right)=r_{delay(s_{delay}\left(S,Q\right))}$$

Note that in this case, the $r_{delay}$ is equal to $d_{score}$ since only the network delay is considered; other characteristics such as datetime for dynamic network delay can be applied.

After defining the three characterization scores, the middleware is able to calculate the SAAQ score and assign each query to the most suitable available server. This configuration phase is supposed to be executed once. It can be re-executed when servers are updated, new devices are added, or new types of queries are integrated into the Ubicomp environment.

### 4.2. Assignment Phase

Once servers, queries, and network delay are characterized, the middleware evaluates the best server for each query received. To do so, two steps are performed as explained in the following.

#### 4.2.1. Step 4: Calculation of the SAAQ Score

The SAAQ score determines if a server (local or remote) or a temporal/permanent server is capable to resolve a specific query whose type is one of those defined in Section 4.1.2. The SAAQ score is calculated by using the server characterization score ($c_{score}$, see Definition 1), the query characterization score ($q_{score}$, see Definition 5), and the delay characterization score ($d_{score}$, see Definition 6).

In order to obtain the SAAQ score ($saaq_{score}$), we first calculate the SAAQ performance score ($saaq_{perf}$), which measures the distance between the server characterization score ($c_{score}$) and the query characterization score ($q_{score}$). This score represents the viability of performing a query in the server without yet considering the delay between the servers. A negative score means that the resources required to attend to a query are greater than the ones of the server; similar query and server resources result in a zero score; while a positive value score represents the server resources are greater than those requested. The SAAQ performance score is formally defined in Definition 7.

**Definition** **7.** **SAAQ Performance Score (${\mathrm{saaq}}_{\mathrm{perf}}$).** *Given a server S and a query Q, the SAAQ performance score, denoted as $saaq_{perf}$, is an integer value, defined as:*

$$saaq_{perf}\left(S,Q\right)=\sum_{i=\{cpu,ram,gpu\}}\left(\left(c_{score}\left(S.r_{i}\right)-q_{score}\left(Q.r_{i}\right)\right)\times q_{score}\left(Q.r_{i}\right)\right)$$

*where: $c_{score}\left(S\right)=\left[S.r_{cpu},S.r_{ram},S.r_{gpu}\right]$ is the server characterization score of S, $q_{score}\left(Q\right)=\left[Q.r_{cpu},Q.r_{ram},Q.r_{gpu}\right]$ is the query characterization score of Q, and i is the CPU, RAM, and GPU.*

Note that the SAAQ performance score takes into account the most demanding resource from the query by multiplying the difference between the server and query scores ($c_{score}-q_{score}$) with the query score ($q_{score}$). For instance, considering two servers, S1 and S2, with characterization scores of $c_{score}\left(S1\right):<3,3,8>$ and $c_{score}\left(S2\right):<8,3,3>$, and a query with $q_{score}\left(Q\right):<1,1,5>$ characterization score, by only calculating the different between both scores, i.e., $c_{score}\left(S\right)-q_{score}\left(Q\right)$, we obtain $saaq_{perf}\left(S1,Q\right)=7$ and $saaq_{perf}\left(S2,Q\right)=7$, and therefore according to the results, any of the servers can attend to the query without identifying which is better. Query *Q* demands more GPU than CPU and RAM resources; thus, S1 is more suitable than S2, since S1 has a $S1.r_{gpu}=8$ ranking value for GPU, while S2 only $S2.r_{gpu}=3$. Multiplying the difference by the query score ($\left(c_{score}\left(S\right)-q_{score}\left(Q\right)\right)\times q_{score}\left(Q\right)$), the results are $saaq_{perf}\left(S1,Q\right)=19$ and $saaq_{perf}\left(S2,Q\right)=-1$, showing that the server S1 is better than S2 for attend to the query.

Considering the motivating scenario described in Section 2, an Image Processing query, whose query cost is $q_{score}\left(Q\right):<6,5,6>$ has a SAAQ performance score of $saaq_{perf}=73$ with respect to Remote Server I; $saaq_{perf}=33$ with respect to Master Server; $saaq_{perf}=-12$ for Server II; $saaq_{perf}=-58$ for the Local Server; while for Robot I and II the score is $saaq_{perf}=-75$. For example, Table 7 summarizes the $saaq_{perf}$ values for an Image Processing query in our motivating scenario.

In order to obtain the $saaq_{perf}$ values into the range [0, 10] and to keep the same scale as the delay characterization score, we normalize the $saaq_{perf}$ values as Equation (1) shows.

(1) $$saaq_{perf\_norm}=10\times \left(saaq_{perf}–min\left(saaq_{perf}\right)\right)/\left(max\left(saaq_{perf}\right)-min\left(saaq_{perf}\right)\right)$$

where $min(saaq_{perf})$ is the minimum and $max(saaq_{perf})$ is the maximum $saaq_{perf}$ scores among values. For instance in our motivating scenario, Robot I or Robot II with $c_{score}:<1,2,1>$ obtain the minimum performance score $min(saaq_{perf})=-75$, solving an Image Processing query ($q_{score}:<6,5,6>$). The Master Server with $c_{score}:<7,8,8>$ obtains the maximum performance score $max(saaq_{perf})=38$, solving a Web query ($q_{score}:<6,4,4>$). After the normalization is applied, Robots I and II with respect to the Image Processing query, obtain a $saaq_{perf\_norm}=0$; the Master Server with respect to a Web query $saaq_{perf\_norm}=10$. Table 8 shows the $saaq_{perf\_norm}$ values for an Images Processing query with respect to the servers in our motivating scenario.

Once the SAAQ performance score is normalized, the SAAQ score can be calculated by adding the delay characterization score ($d_{score}$). Definition 8 formalizes our SAAQ score.

**Definition** **8.** **SAAQ Score (${\mathrm{saaq}}_{\mathrm{score}}$).** *Given a server S and a query Q, the server ability to resolve a query, denoted as $saaq_{score}$, is defined as:*

$$saaq_{score}\left(S,Q\right)=\alpha \times saaq_{perf\_norm}\left(S,Q\right)-\beta \times d_{score}\left(S,Q\right)$$

*where α and β define the importance of the delay characterization score, which are user-preference parameters; such that α + β = 1.*

Table 9 presents the $saaq_{score}$ values for our motivating scenario, considering α=0.80 and β=0.20. Higher values mean that the server is more powerful to attend to the query; however, computational resources can be wasted by a simple query attended on high performance servers.

The following section describes how to select the most adequate server for each query.

#### 4.2.2. Step 5: Assignment Query Process

In order to assign a query to a specific server, it is necessary to determine from what baseline a $saaq_{score}$ value represents a suitable assignment scenario. The SAAQ performance score ($saaq_{perf}$, see Definition 7) determines if a server has the resources to attend to a query, deciding a positive scenario when this score is equal to or greater than zero, i.e., $saaq_{perf}\in \left\{0,Q^{+}\right\}$. Thus, $saaq_{perf}=0$ is our baseline to determine a correct assignment. Our approach for calculating the $saaq_{perf}$ (see Definition 7) is universal and can be applied to any scenario. Since the $saaq_{perf}$ is normalized between 0 and 10 to obtain $saaq_{perf\_norm}$, our baseline has to be normalized as well using the same $min(saaq_{perf})$ and $max(saaq_{perf})$ values of the particular context, which in our motivating scenario are $min(saaq_{perf})=-75$ and $max(saaq_{perf})=38$ (see Section 4.2.1), respectively. After normalization, i.e., $10\times \left(baseline-min\left(saaq_{perf}\right)\right)/max\left(saaq_{perf}\right)-min\left(saaq_{perf}\right))$, the normalized motivating-scenario baseline value is 5.309. In Table 9, the green marked scores are the values that comply with the normalized baseline, i.e., score greater or equal to 5.309.

To select the most suitable server from the ones that are able to resolve the queries, we adopt a philosophy of the use of minimum resources, which allows a lower energy consumption and the availability of high-performance servers for complex queries that really need it, i.e., while the $saaq_{score}$ value is closer to the normalized baseline value, the server is more suitable than others that could also satisfy the query. Table 9 shows the most suitable servers which are marked with an asterisk. For example, for Images Processing queries, the most suitable server is the Master Server, while for Synchronization queries are Robot I and Robot II.

Note that Remote Server I ($s_{score}\left(RSI\right):<10,10,10>$) is not the most suitable server for any of the queries even if it is the most powerful server. This server can attend to more complex queries than the ones defined in this work, saving energy, and also being available to process the requests when the most suitable servers are busy. Cases in which none of the servers are able to attend to a query due to a $saaq_{score}$ less than the normalized baseline value, the same philosophy of minimum resources is applied, selecting the server with the $saaq_{score}$ closer to the baseline (under the baseline). Using the $saaq_{score}$ value, a load balancer can make decisions not only considering the load on servers but also the server ability to resolve the query for better response time. By following this methodology, location awareness, as well as energy awareness are fulfilled due to a consideration of network delays, the capacity to serve, and the categorization of services and servers.

The following section describes the experiments in order to evaluate our proposal.

## 5. Experimental Evaluation

To evaluate and validate the SAAQ-based methodology, we implemented a distributed server architecture (middleware), considering the $saaq_{score}$ value as a condition of query assignment, as well as the CPU utilization of servers. In the following sections, we describe the main aspects considered for the experimental evaluation.

### 5.1. Query Implementation

As we presented in Section 4.1.2, we consider four categories of queries: (i) Multimedia; (ii) Localization; (iii) Web Services; and (iv) Information Management Services, from which we propose six query types. In order to evaluate our proposal, the six queries are implemented as follows:

- **Images Processing query**: Client sends a 1.8 MB picture as query data and receives the same image as response from the server.
- **Web query:** The text of the Uniform Resource Locater type (URL) “www.rutas.com.pe” (accessed on 1 March 2021) is received as a response.
- **Word Processing**: A text about a historical review of the city of Arequipa, Peru, of 1842 bytes is sent, and the same text is received as response data once it is processed.
- **Synchronization query**: The Linux “date” command is executed on the assigned server system, and the date and time information extracted from the system are stored in a 29-byte text that is sent as response data to the client.
- **Localization by Images**: A 1.6 MB image is sent and a random geographic location is received in response.
- **Localization by IP address**: In this type of query, the client sends its IP address and receives a random geographic location as a response.

Table 10 summaries our implemented queries. The size of images for Images Processing and Localization by image queries are 1.8 MB and 1.6 MB, respectively, since we have considered the Pepper robot, whose hardware specification indicates a 5 Megapixel camera; thus, it produces between 1.5 MB and 1.9 MB size pictures. This robot is considered an IoT server due to its limited hardware [54].

### 5.2. Architecture Implementation: Servers

A total of 12 servers were hired from the company Digital Ocean, a North American company that provides virtual, private, and cloud services, headquartered in New York City [55]. Table 11 shows a summary of the hardware aspects of each team hired and selected for this phase.

The hardware configuration of clients (high performance) was selected considering that thousands of simultaneous connections will be simulated from the same physical equipment; therefore a large amount of RAM memory and numbers of cores of CPU are required (eight dedicated cores, 16 GB RAM). Due to that, the creation time of these connections is as fast and simultaneous as possible.

An intermediate power server for the Master Server was selected, since it is in charge of distributing the work and also processing when it is the case. The local and remote servers were selected as low-power servers. All clients and servers have an SSD of 25 GB of capacity and run Ubuntu 20.04 LTS.

### 5.3. Architecture Implementation: Protocol and Configuration

The programming language used for the implementation of the current version of the middleware is C language. The C language presents the best performance in terms of energy consumption, execution times, and memory occupation [48,56].

#### 5.3.1. Communication Protocol

In the current implementation of the middleware, communication sockets were defined under the Stream Control Transmission Protocol (SCTP). SCTP is defined as a transport layer protocol in the Open Systems Interconnection (OSI) model, which allows the transmission of several data streams between two end points when the connection in the network is established at the same time [57].

Based on the state of the art, it was determined that the SCTP protocol is the most recommended in terms of performance, jitter (delay fluctuation), delay, and packet loss. It is also considered the most adjusted protocol for the multimedia data transmission [57,58,59,60].

#### 5.3.2. Configuration

All servers in the test scenario were configured to ensure optimal performance of the architecture, considering the following parameters:

- **Thread numbers:** The first parameter to be defined is the number of threads in the threads pool of each server in the communication socket programmed in C language. This pool of threads establishes the number of simultaneous connections that servers can handle. It was determined by experimentation, obtaining the best performance with 250 threads for the Master Server and 150 threads for the other servers, since the Master Server has better hardware than the other ones.
- **File descriptors:** The number of file descriptors defined by default (1024) is insufficient when it is required to process thousands of queries and processes simultaneously; thus, to avoid the known error *too many file opens*, it is pertinent to set a number greater than the default value. It was experimentally determined for this scenario that the optimal number of file descriptors to allow servers to process thousands of queries simultaneously without producing errors is 8192; this parameter is set with the Linux command “ulimit -n 8192”.
- **Libraries:** Library “netinet/sctp.h” must be installed to run the server sockets under the SCTP, and include all the data handling and connection characteristics of this communication protocol. Its installation was carried out through the command “sudo apt install libsctp-dev” and is executed in the following way “gcc mysocket.c –o mysocket.out –lsctp”. Library “pthread.h” is necessary to run the C program, from the multithreaded client or server socket, using command “gcc mysocket.c –o mysocket.out –lpthread”. Additionally, tool “glxinfo”, by the command “apt-get install mesa-utils” was executed, where the MESA library was installed, which provides a generic implementation of OpenGL, which is an Application Programming Interface (API) cross-platform graphics that specifies a standard software interface for three-dimensional (3D) graphics processing hardware [61,62]. In this sense, to extract the information from the computer’s graphic card, the command “glxinfo ∣ grep OpenGL”, when the first connection of each SE with the SM was established.

### 5.4. Experiments and Results

This section describes the tests performed to evaluate the response times of processing the queries as well as CPU consumption of servers, and thus, the performance of the methodology developed. An efficient load balancer is able to distribute tasks without stressing a server (cpu usage 100%). Experiments are related to location awareness (different client location), energy awareness (allocation of necessary computational resources), and automatic categorization of services and servers (using our proposed rankings).

Tests are performed considering the delay times between Client 1 (New York) and the servers presented in Table 12 which are the average of five repetitions. The delay time between Client 1 (New York) and the Master Server (San Francisco) is smaller than the one between Client 1 and Remote Server II (Singapore), since the distance of the latter is greater. Experiments were performed during March 2021.

The α=0.800 and β=0.200 values for $saaq_{perf\_norm}$ and $d_{score}$, respectively, are used to calculate the $saaq_{score}$ value. The normalized baseline value for this scenario is 5.380 as we explain the calculation in Section 4.2.1. Table 13 shows the $saaq_{score}$ values, where the ones marked as green are the suitable servers for the corresponding query.

**Figure 3:**
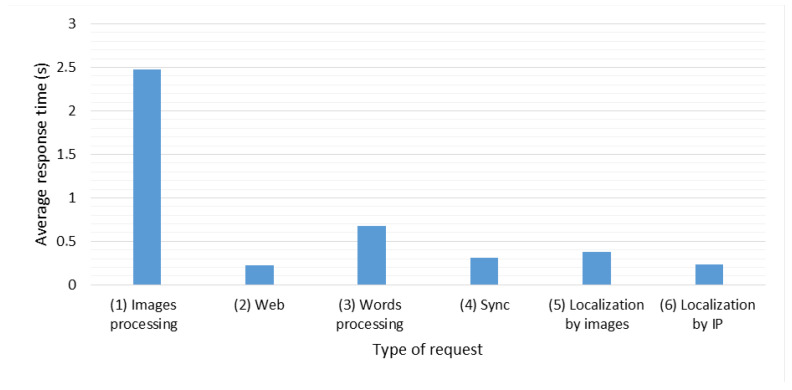
Average response times with respect to the type of request.

**Test 1: Average Response Time for each type of Query.** The objective of this test is to determine the average response times of five repetitions for each type of query generated from Client 1. Figure 3 shows the results obtained in this test. The query with the highest average response time is the Image Processing query (2.4767 s), since it sends and receives an image of 1.8 MB, followed by the Word Processing query with response time in 0.6752 s. The query with the shortest response time is the Web query (0.2252 s). The Synchronization query performed in 0.3074 s; Location by Image query was executed in 0.3752 s, while Location by IP address was executed in 0.2303 s in average. Five of the six types of queries obtained an average response time less than 1 s. Note that the times obtained were affected by the network delay between the servers, already specified in Table 12.

**Test 2: Performance for Simultaneous Connections.** With this test, we determine the performance of the developed architecture, when 20, 100, 500, 1000, and 2000 simultaneous connections of each type of query are generated by Client 1 (six types of queries), including an extra one which refers to a random type query (seven types of queries in total) for a more real scenario where several clients perform different type of request. The scenario used in this test is the same as *Test 1*, under the same $saaq_{score}$ and delay conditions. In order to simplify the name of queries, we renamed the Image processing query as *Type 1*, Web queries as *Type 2*, Word Processing query as *Type 3*, Synchronization query as *Type 4*, Localization by Images query as *Type 5*, Localization by IP address as *Type 6*, while random query (any of the six types of queries) as *Type 7*.

**Figure 4:**
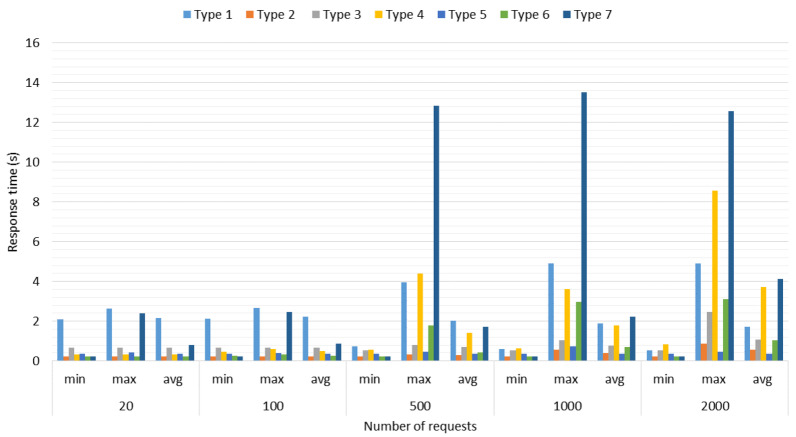
Response times with respect to the number of connections.

Figure 4 shows the maximum, minimum, and average response times obtained in this test. Response times increase by staggered increasing the number of connections that are generated regardless of the type of query. This effect is produced due to the queued which is generated in the Distribution Layer made up of the Master Server, the more connections are generated, the longer queries take a long time to be assigned and answered. Queuing becomes noticeable after 500 connections; below this number, queuing has no effect on response times; it can be observed especially through the maximum time in Figure 4.

**Figure 5:**
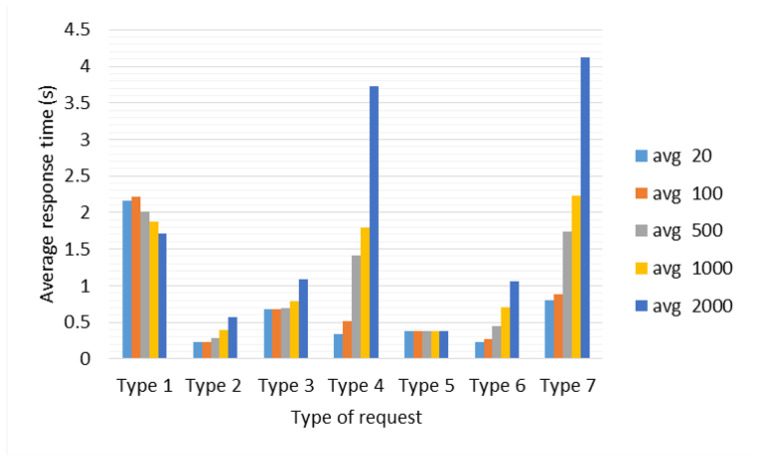
Average response times with respect to the type of request from 20 until 2000 requests.

**Figure 6:**
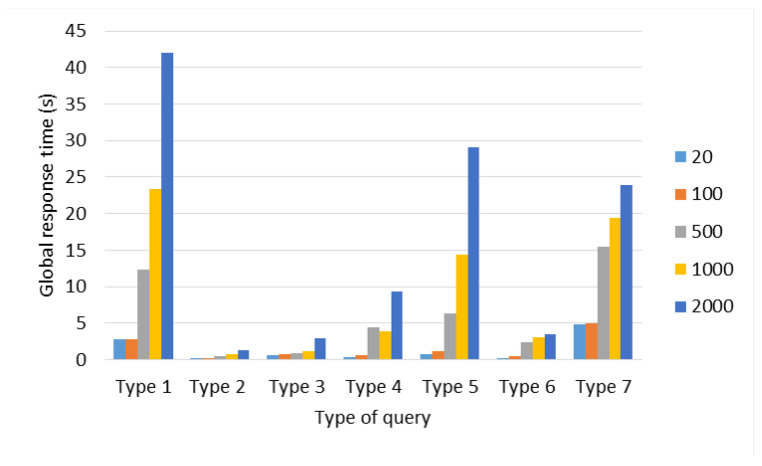
Global response times with respect to the type of request.

On the other hand, *Type 4* (Synchronization) and *Type 7* (Random) queries, as can be seen in Figure 4, are the ones that present the greatest increase in maximum response times because of their dynamic allocation, i.e., a set of queries can be served by the Master Server, while another set must be assigned to the Local or Remote Servers and implies that the delay in terms of computational processing has a greater impact on global (time to response of all queries) and average response time (see Figure 5 and Figure 6).

The *Type 4* (Synchronization) query, unlike the rest of the queries, generates a system call with the command “date”, therefore, the more queries of this type are made, there will be more calls to the system, which represents a greater delay in terms of computational processing in comparison to the rest of the queries, although by itself this type of query does not require high computational resources. For *Type 5* (Localization by Images) query (see Figure 5), the average response time does not show a notable variation when scaling the number of connections; however, in Figure 6, from 500 connections, *Type 1* (Images Processing), *Type 5* (Localization by Images), and *Type 7* (Random) queries start to significantly increase their global response times, due to queuing in the Distribution Layer.

The factors that affect the response times in queuing at the Distribution Layer are the number of threads in the server thread pool and the number of CPU cores. The number of threads defines, at the software level, how many tasks the server must solve simultaneously, and at the hardware level, the cores tell us how many tasks at the physical level the equipment is capable of solving. However, the server tries to grant the limited resource of the number of CPU cores fairly to all the threads waiting to be executed in their entirety in order to resolve them pseudo-simultaneously. For this reason, this pseudo-simultaneity generates a delay, and since in the Master Server the number of threads was defined as 250 and in the rest of the servers as 150, then, when it receives more than 250 connections it begins to scale significantly the global response times. Note that the Master Server distributes tasks efficiently between itself and the servers (local or remote), i.e., the Master Server also responds to queries from clients.

**Figure 7:**
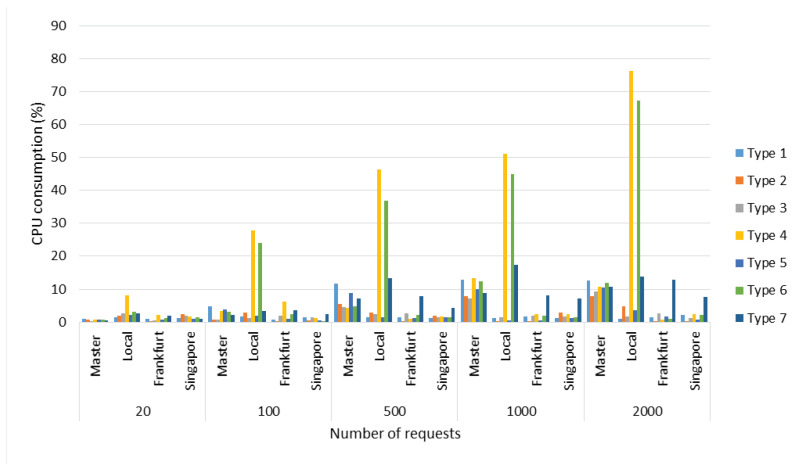
Average CPU consumption with respect to the numbers of connections.

Regarding CPU consumption, in Figure 7, the server with the highest CPU activity is the Local Server for query *Type 4* (Synchronization) and *6* because it is the most suitable in terms of $saaq_{score}$ (up to 80% approximately, see Table 13). Additionally, it can be observed that from 500 connections of *Type* 7 (random queries), activity was registered in the Remote servers, which implies that the load balancer (Master Server) perceived greater activity in the Local Server, and therefore decided to assign part of the tasks to the rest of the Remote Servers. It is important to highlight that the metadata information is sent every 4 s to the Master Server in order to know the state of the server (each Local and Remote Servers send metadata information to the Master Server), for this reason, the longer the time the architecture was stressed, the more efficient the load balance was.

The performance obtained from the middleware in terms of response time is related to the capacity of the master server to receive and attend to the queries. Queuing becomes noticeable for 500 and more concurrent connections, since the pool of threads for the master server was established to 250 by experimentation according to the resources of this server. Different hardware configurations require changes in the number of threads of the server that it can handle simultaneously.

**Test 3: Performance by different Client Locations.** The objective of this test is to determine the impact of the client location from where the queries are generated and additionally to verify if the existence of a server close to the client contributes to obtain better response times as part of the proposed task assignment scheme.

**Figure 8:**
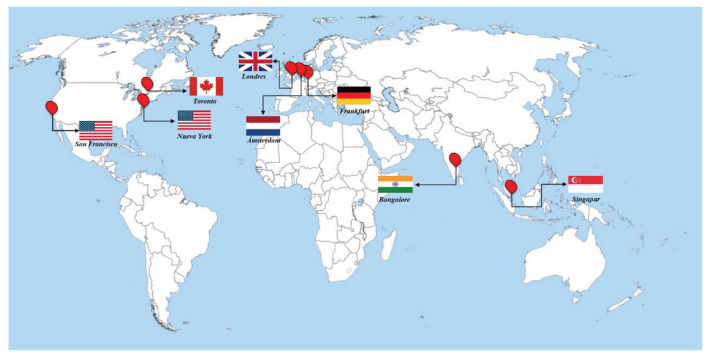
Location of clients available in digital ocean.

For this test, there are seven evaluating scenarios since seven different geographical locations of servers are available in the Digital Ocean service (see Figure 8). Due to only *Type 4* (Synchronization) and *Type 6* (Localization by IP address) queries being able to be performed by the Local and Remote Servers according to the $saaq_{score}$ (see Table 13), their $saaq_{score}$ values are modified to comply (as shown in Table 14 ) and therefore evaluate the client location performance.

For O1 and O2 scenarios, the $saaq_{score}$ values are shown in Table 15. According to this table, the Master Server is able to serve all the types of queries, as can be seen in the green boxes, and for *Type 4* (Synchronization) and *6* (Localization by Images) queries, the first candidate is the Local Server in San Francisco, followed by the Master Server, then Remote Server I in Frankfurt, and lastly Remote Server II in Singapore.

For O3, O4, and O5 scenarios, the $saaq_{score}$ values described in Table 16 are considered, where the Master Server is also capable of handling all types of queries. In the case of *Type 4* (Synchronization) and *6* queries, the first candidate is Remote Server I in Frankfurt, the second is the Master Server, followed by the Local Server in San Francisco and lastly Remote Server II in Singapore.

For scenarios O6 and O7, the $saaq_{score}$ values are described in Table 17, where the Master Server is capable of handling all types of queries as well. For *Type 4* (Synchronization) and *Type 6* (Location by IP address) queries, the first candidate is Remote Server II in Singapore, followed by Master Server, then Local Server in San Francisco, and lastly Remote Server I in Frankfurt.

For this test, 2000 connections were generated with types of random queries (*Type 7*).

**Figure 9:**
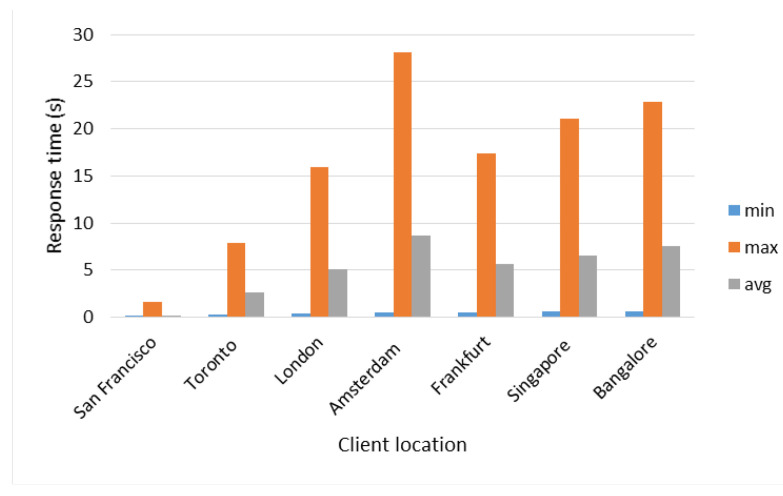
Minimum and maximum response times according to client location.

**Figure 10:**
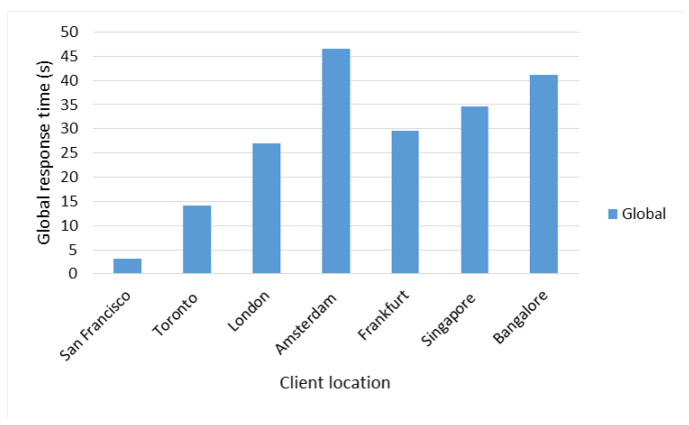
Global response times according to client location.

Regarding the results obtained in this test, it can be observed in Figure 9 and Figure 10 that the shortest average and total response times were obtained with the client located in the city of San Francisco, while the highest average and total response times were obtained with the client located in the city of Amsterdam.

**Figure 11:**
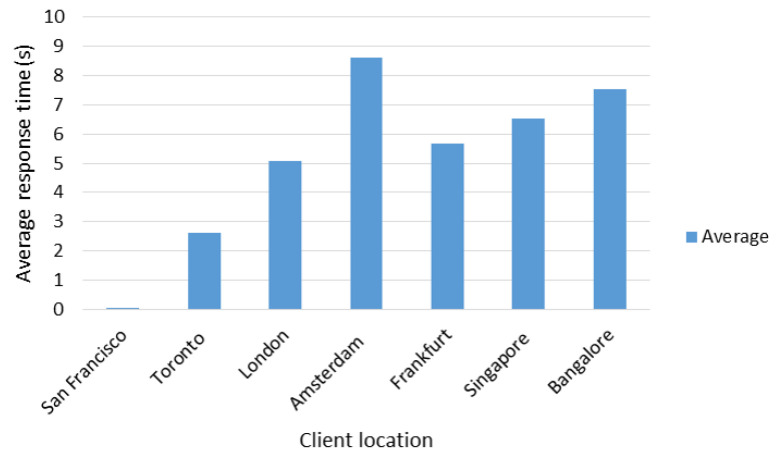
Average response times according to client location.

For the average response time, a behavior similar to the ones of the total times was observed (see Figure 11), where the longest time was obtained with the client in the city of Amsterdam and the lowest in the city of San Francisco.

Table 18 shows the network delay between each client and the Master Server, and between clients and the closest suitable server (Local Server, Remote Server I, or Remote Server II). Given this information and in addition to that shown in previous figures, it was determined that the network delay has a considerable effect on the response times according to the location of the client, as well as the distance between the Master Server and the nearest suitable server.

In the case of the client in the city of San Francisco, the fact of having a delay of only 0.5 milliseconds (ms) with respect to the Master Server and the Local Server (see Table 18, row 2), both also located in San Francisco, guaranteed to obtain average response time of 19 ms approximately.

In the case of clients in Singapore and Bangalore, despite the fact that the former had a greater delay with respect to the Master Server, it obtained better average response times due to the fact that it had an remote server (Remote Server 2) located in the same city of Singapore as opposed to the client in Bangalore which had a delay of 33.67 ms with respect to the nearest remote server. The client in Amsterdam obtained the highest average response time of the entire test, given that its delay with respect to the Master Server was the highest in the entire test (193.30 ms) and with respect to its closest remote server (Remote Server I—158.56 ms), it was also the highest among clients located in Europe. By not having a remote server in the same location as the client, response times are high.

**Figure 12:**
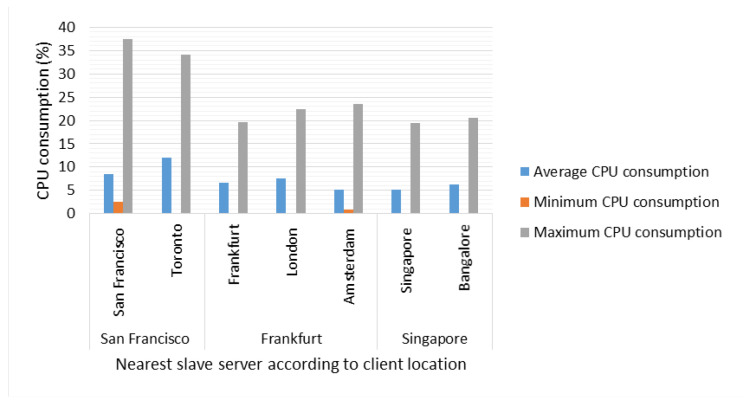
Average CPU consumption by client location.

Finally, with respect to CPU consumption, a record is taken of the CPU activity in the server closest to the clients in each test. As can be seen in Figure 12, the Local Server in San Francisco registered the highest average CPU activity with 13% for the client located in Toronto. In Europe, for the client, the remote server in Frankfurt recorded the highest CPU activity with 7% average usage, while in Asia, in the scenario in Bangalore, the remote server in Singapore, recorded the highest activity with 11% average CPU occupancy.

**Discussion:** Regarding the impact of delay in response times, it was partially determined by the distance between the Master Server, the remote servers, and the clients. Generating the queries in the same location of the Master Server and Local Server (San Francisco), the average response time did not exceed 70 ms. That is, the incidence of delay was significantly reduced, leading us to determine that the process of load balancing, query allocation, and the SCTP protocol ensured fast response times. On the other hand, in the generation of queries from locations that are distant from the Master Server, a factor that considerably improves response times is having a remote server in the same location of the client, as happened in the case of Singapore and Frankfurt. This determines that the strategy for assigning queries to the remote server closest to the client was optimal and fulfilled its objective of reducing response times.

## 6. Conclusions and Future Works

In this work, we proposed a characterization method for distributed servers in Ubicomp environements, where the computational resources of servers are taken into account for better response times. A heterogeneous architecture, with devices with different capacities acting as servers and clients at the same time, makes traditional load balancers not suitable for this scenario.

Preliminary experiments under the scenarios where distance and workload are considered, demonstrated the viability of our proposal. All generated queries reported having received their corresponding responses in all the tests carried out, which determines that both the SCTP protocol and the middleware of communication developed under all scenarios guaranteed the integrity of the query and response data.

Our proposal was able to distribute the loading, showing during experiments a maximum use of CPU around 80% for the Local Server and distributing the queries to remote servers when this value was reached (2000 simultaneous requests). However, since metadata information about the remote servers state are sent every 4 s, an optimal real-time assignment is not possible since if the Master Server is able to assign thousands of queries in less than 4 s, all queries will be attended to by the most suitable server, overloading it.

We are currently working on a server-state prediction method based on the use of server resources and the complexity of the queries for a better query assignment. The size of data sent by the queries is another factor to be included. Moreover, a dynamic network delay will be integrated, considering the network congestion during the assignment process. Experiments for a correct delay network impact on the $saaq_{score}$, i.e., α and β values, will also be evaluated in the future.

## Figures and Tables

**Table 1:** Proposed CPU Score.

CPU Example	CPU Score $s_{\mathrm{cpu}}$	Raking $r_{\mathrm{cpu}}$	Reference Architecture CPU	
			CPU Score	Server
AMD Threadripper 3990X	$s_{cpu}$ > 10,000	10	35,225	Remote Server I
AMD Threadripper 3970X	6000 < $s_{cpu}$ ≤ 10,000	9		
Procesador Intel Xeon Platino 8160	2000 < $s_{cpu}$ ≤ 6000	8		
Intel Core i9-9900x	700 < $s_{cpu}$ ≤ 2000	7	880	Master Server
Intel i7 8700	281 < $s_{cpu}$ ≤ 700	6		
Intel i7 7800X	115 < $s_{cpu}$ ≤ 281	5	202	Remote Server II
AMD Ryzen 5 1400	100 < $s_{cpu}$ ≤ 115	4		
AMD A10-9700	90 < $s_{cpu}$ ≤ 100	3	91.2	Local Server
Intel Core i7-8650U (2.7 GHz)	83.2 < $s_{cpu}$ ≤ 90	2		
Intel i3 2330 M	$s_{cpu}$ ≤ 83.2	1	30.56	Robot A and B

**Table 2:** Proposed RAM Score.

RAM Score $s_{\mathrm{ram}}$	Ranking $r_{\mathrm{ram}}$	Reference Architecture RAM	
		RAM Score	Server
$s_{ram}\geq 128$	10	128 GB	Remote Server I
100 < $s_{ram}$ < 128	9		
64 < $s_{ram}$ ≤ 100	8	64 GB	Master Server
32 < $s_{ram}$ ≤ 64	7–6		
16 < $s_{ram}$ ≤ 32	5	16 GB	Remote Server II
8 < $s_{ram}$ ≤ 16	4		
4 < $s_{ram}$ ≤ 8	3	4 GB	Local Server
2 < $s_{ram}$ ≤ 4	2	2 GB	Robot A and B
$s_{ram}$≤ 2	1		

**Table 3:** Proposed Score for GPU.

GPU Example	GPU Score $s_{\mathrm{gpu}}$	Ranking $r_{\mathrm{gpu}}$	Reference Architecture GPU	
			GPU Score	Server
Nvidia RTX 3090	$s_{gpu}$ < 40.8	10	40.80	Remote Server I
Nvidia GTX 950	28.8 ≤ $s_{gpu}$< 40.8	9	-	-
AMD RX 590	12 ≤ $s_{gpu}$< 28.8	8	12	Master Server
Nvidia GeForce MX250	6.4 ≤ $s_{gpu}$< 12	7	-	-
AMD Radeon HD 6670	1.2 ≤ $s_{gpu}$ < 6.4	6	-	-
Nvidia GeForce GTX 280	0.64 ≤ $s_{gpu}$<1.2	5	0.64	Remote Server II
Nvidia Quadro FX 880M	0.56 ≤ $s_{gpu}$ < 0.63	4	-	-
Intel HD 5500 (Mobile 0.95 GHz)	0.51 ≤ $s_{gpu}$ < 0.56	3	0.52	Local Server
ATI Mobility FireGL V5700	0.15 ≤ $s_{gpu}$ < 0.51	2	-	-
NVIDIA GeForce 7150M + nForce 630M or Rendering processes appointed to MESA Library	$s_{gpu}$< 0.15	1	0	Robot I and II

**Table 4:** Scores for the reference architecture in the motivating scenario.

Server	Characterization Score $c_{\mathrm{score}}$
Remote Server I	<10, 10, 10>
Master Server	<7, 8, 8>
Remote Server II	<5, 5, 5>
Local Server	<3, 3, 3>
Robot I and Robot II	<1, 2, 1>

**Table 5:** Query Characterization Score for the motivating scenario.

Service	Type of Query	Query Characterization Score ($q_{\mathrm{score}}$)
Multimedia Service	(1) Image Processing	<6,5,6>
Web Service	(2) Webiste and Apps Accessing	<6,4,4>
Information Management Service	(3) Words Processing (4) Syncronization (updating data)	<6,5,2> <1,1,1>
Localization Service	(5) Localization by Images (6) Localization by IP address	<6,4,5> <3,2,1>

**Table 6:** Cost of network delay between servers or client-servers.

Delay Network Score (ms) $\left(s_{\mathrm{delay}}\right)$	Ranking ($r_{\mathrm{delay}}$)	Reference Architecture
0– 20	0	Master Server
21–40	1	Robot I/Robot II/Local Server
41–60	2	-
61–80	3	-
81–100	4	-
101–120	5	-
121–140	6	-
141–160	7	-
161–180	8	Remote Server I
181–200	9	Remote Server II
200+	10	-

**Table 7:** SAAQ performance scores for our Motivating Scenario.

**Server Score**	**Remote** **Server I** **[10, 10, 10]**	**Master** **Server** **[7, 8, 8]**	**Remote** **Server II** **[5, 5, 5]**	**Local** **Server** **[3, 3, 3]**	**Robots** **[1, 2, 1]**
**Query Cost**					
**Images** **Processing** **[6, 5, 6]**	73	33	−12	−58	−75

**Table 8:** $saaq_{perf\_norm}$ values for our Motivating Scenario.

**Server Score**	**Remote** **Server I** **[10, 10, 10]**	**Master** **Server** **[7, 8, 8]**	**Remote** **Server II** **[5, 5, 5]**	**Local** **Server** **[3, 3, 3]**	**Robots** **[1, 2, 1]**
**Query Cost**					
**Images** **Processing** **[6, 5, 6]**	10	7.290	4.250	1.140	0

**Table 9:** $saaq_{score}$ values for our motivating scenario.

**Server Score**	**Remote** **Server I** **[10, 10, 10]**	**Master** **Server** **[7, 8, 8]**	**Remote** **Server II** **[5, 5, 5]**	**Local** **Server** **[3, 3, 3]**	**Robots** **[1, 2, 1]**
**Query Cost**					
**Images** **Processing** **[6, 5, 6]**	9.072	7.645 *	2.860	2.053	0
**Syncronization** **[1, 1, 1]**	5.821	6.725	4.559	5.734	5.380 *
**Localization** **by Images** **[6, 4, 5]**	9.072	7.928	3.568	3.044	1.203
**Localization** **by IP address** **[3, 2, 1]**	7.166	7.504	4.842	5.592 *	4.884
**Web Query** **[6, 4, 4]**	9.007	8 *	3.851	3.469	1.769
**Words** **processing** **[6, 5, 2]**	8.511	7.645 *	3.709	3.185	1.981

The green marked scores are the values that comply with the normalized baseline; The servers marked with an asterisk are the most suitable for the type of query.

**Table 10:** Used data set in experiments.

Type of Query	Query Data Type	Size of Query Data (Bytes)	Response Data Type	Size of Response Data (Bytes)
Image processing	Image	1.800.000	Image	1.800.000
Web query	Text	1	Text	15
Words processing	Text	1.842	Text	1.842
Syncronization	Text	1	Text	29
Localization by images	Image	1.668.636	Text	30
Localization by IP address	Text	1	Text	30

**Table 11:** Hired servers used during experiments.

Role	CPU State	Location	CPU Core N°	RAM Memory	Price Per Month	Server Characteri. Score ($c_{\mathrm{score}}$)
Master Server	Share	San Francisco	8	16 GB	80$	[7, 5, 8]
Local Server	Share	San Franciso	1	2 GB	10$	[5, 1, 1]
Remote Server I	Share	Frankfurt	1	2 GB	10$	[5, 1, 1]
Remote Server II	Share	Singapore	1	2 GB	10$	[5, 1, 1]
Client 1	Dedicated	New York	8	16 GB	160$	[7, 5, 1]
Client 2	Dedicated	Toronto	8	16 GB	160$	[7, 5, 1]
Client 3	Dedicated	London	8	16 GB	160$	[7, 5, 1]
Client 4	Dedicated	San Francisco	8	16 GB	160$	[7, 5, 1]
Client 5	Dedicated	Amsterdam	8	16 GB	160$	[7, 5, 1]
Client 6	Dedicated	Frankfurt	8	16 GB	160$	[7, 5, 1]
Client 7	Dedicated	Bangalore	8	16 GB	160$	[7, 5, 1]
Client 8	Dedicated	Singapore	8	16 GB	160$	[7, 5, 1]

**Table 12:** Delay times between Client 1 and servers.

From (Client 1)	To	Delay (ms)
New York	San Francisco (Master Server)	75.7600
New York	San Francisco (Local Server)	75.5479
New York	Frankfurt (Remote Server I)	84.2000
New York	Singapore (Remote Server II)	247.7850

**Table 13:** Assignment query process based on $saaq_{score}$.

Server	Master	Remote I	Remote II	Local	Renamed
Query					
Images processing	7.645	−0.055	−0.455	0	Type 1
Web and apps	6.725	1.785	1.385	1.769	Type 2
Word processing	7.928	1.219	0.819	1.203	Type 3
Synchronization	7.504	5.500	5.600	5.380	Type 4
Localization by images	8	1.219	0.819	1.203	Type 5
Localization by Ip address	7.645	5.500	5.600	5.380	Type 6

The green marked scores are the values that comply with the normalized baseline.

**Table 14:** Client location and most suitable servers for *Type 4* (Synchronization) and *Type 6* (Localization by IP address) queries.

Scenario	Client Location	Most Suitable Server
**O1**	Client 2 (San Francisco—USA)	Local Server (San Francisco—USA)
**O2**	Client 3 (Toronto—Canada)	Local Server (San Francisco—USA)
**O3**	Client 4 (Frankfurt—Germany)	Remote Server 1 (Frankfurt—Germany)
**O4**	Client 5 (London—United Kingdom)	Remote Server I (Frankfurt—Germany)
**O5**	Client 6 (Amsterdam—Netherlands)	Remote Server I (Frankfurt—Germany)
**O6**	Client 7 (Singapore—Singapore)	Remote Server II (Singapore—Singapore)
**O7**	Client 8 (Bangalore—India)	Remote Server II (Singapore—Singapore)

**Table 15:** $Saaq_{score}$ values for Scenarios O1 and O2.

Server	Master	Remote I	Remote II	Local
Query				
Images processing	7.645	−0.055	−0.455	0
Web and apps	6.725	1.785	1.385	1.769
Word processing	7.928	1.219	0.819	1.203
Synchronization	7.504	7.600	7.700	5.380
Localization by images	8	1.219	0.819	1.203
Localization by Ip address	7.645	7.700	7.750	5.380

The green marked scores are the values that comply with the normalized baseline.

**Table 16:** $Saaq_{score}$ values for scenarios O3, O4, and O5.

Server	Master	Remote I	Remote II	Local
Query				
Images processing	7.645	−0.055	−0.455	0
Web and apps	6.725	1.785	1.385	1.769
Word processing	7.928	1.219	0.819	1.203
Sync	7.504	5.380	7.700	7.600
Localization by images	8	1.219	0.819	1.203
Localization by Ip address	7.645	5.380	7.750	7.700

The green marked scores are the values that comply with the normalized baseline.

**Table 17:** $saaq_{score}$ values for scenarios O6 and O7.

Server	Master	Remote I	Remote II	Local
Query				
Images processing	7.645	−0.055	−0.455	0
Web and apps	6.725	1.785	1.385	1.769
Word processing	7.928	1.219	0.819	1.203
Sync	7.504	7.700	5.380	7.600
Localization by images	8	1.219	0.819	1.203
Localization by Ip address	7.645	7.750	5.380	7.700

The green marked scores are the values that comply with the normalized baseline.

**Table 18:** Average response time and network delay of third test.

Client Location	Average Response Time (ms)	Client- Master Server Network Delay (ms)	Master- Nearest Suitable Server Network Delay (ms)	Client- Nearest Suitable Server Network Delay (ms)
**San Francisco**	19.1	0.6578	0.5980	0.6016
**Toronto**	2602.3	59.0250	0.5611	59.1670
**Frankfurt**	5654.6	157.4379	158.5471	0.5799
**London**	5074.5	144.6109	158.5580	13.5938
**Amsterdam**	8624.4	193.2961	158.5583	18.6036
**Singapore**	6533.0	186.0983	186.5254	0.4039
**Bangalore**	7518.7	173.2195	186.5140	33.6762

## Data Availability

Data available in a publicly accessible repository.
